# Occupational Performance Coaching With Parents to Promote Community Participation of Young Children With Developmental Disabilities: Protocol for a Feasibility and Pilot Randomized Control Trial

**DOI:** 10.3389/fped.2021.720885

**Published:** 2021-11-05

**Authors:** Chi-Wen Chien, Yuen Yi Cynthia Lai, Chung-Ying Lin, Fiona Graham

**Affiliations:** ^1^Department of Rehabilitation Sciences, The Hong Kong Polytechnic University, Kowloon, Hong Kong SAR, China; ^2^Institute of Allied Health Sciences, College of Medicine, National Cheng Kung University, Tainan, Taiwan; ^3^Rehabilitation Teaching and Research Unit, Department of Medicine, University of Otago, Wellington South, New Zealand

**Keywords:** occupational performance coaching, community participation, children with developmental disability, study protocol, randomized control trial (RCT) designs

## Abstract

**Background:** High rates of restricted community participation have been reported in young children with developmental disabilities. Occupational performance coaching (OPC), grounded in self-determination theory, aims to facilitate children's participation in life situations through coaching parents. However, there have been limited randomized controlled trials demonstrating the efficacy of OPC, especially with a specific focus on children's community participation. The proposed study is the first step in evaluating the feasibility and acceptability of conducting a pilot randomized controlled trial of OPC in Hong Kong and testing its initial efficacy (in comparison to parent consultation) in promoting children's community participation.

**Method/Design:** A feasibility and pilot double-blind randomized controlled trial will be undertaken. Fifty children aged 6 years or below with developmental disabilities and their parents will be recruited from early intervention centers and/or through social media in Hong Kong. Parents will be randomly assigned to receive OPC or consultation, and will be blinded to group allocation. Outcomes will be assessed by blinded assessors at baseline, pre-intervention, post-intervention, and follow-up. Predetermined success criteria will be used to assess the feasibility of the trial. Qualitative interviews will be conducted with parents to explore the acceptability and perceived impact of OPC.

**Discussion:** This trial will test whether the study protocol and OPC are feasible and acceptable, as well as assess the initial efficacy of OPC to obtain effect size estimates. The results of the trial will inform future preparations for conducting a full-scale efficacy trial of OPC.

**Trial Registration:** ClinicalTrials.gov, U.S. National Library of Medicine, National Institutes of Health (#NCT04796909), Registered on 15th March 2021.

## Introduction

Foundations for lifelong health begin in the first 6 years of a child's life through participation in everyday activities (1). For children with developmental disabilities (DD), this is a challenge well documented in the World Report on Disability (2) regarding the barriers they face in fully participating in society. DDs are health conditions that develop at birth and can include mild to profound intellectual disability, impaired speech, emotional dysregulation, and/or motor dysfunction (3, 4). These health conditions often have significant effects on children's abilities to take part in community activities (5–7), which may contribute to an increased risk of health problems (e.g., depression or social isolation) prevalent among children with DD and their families (8–11).

Community participation gives children valuable contexts in which they can learn skills, make friends, foster independence, and develop their sense of purpose by engaging in cultural, artistic, and recreational activities with similar-aged peers living in the same community (12, 13). Community participation is important for children with DD, particularly in the preschool years; this is a critical period for their development, in which their emerging cognitive, speech, social, or motor functions may be compromised by the disability (14, 15). Despite the importance of community participation, studies have reported that young children with DD participate less often in community activities and are less involved in age-related activities compared to their peers (16–19). For children with DD in Hong Kong, their community participation patterns appear to be worse than those of children in other countries (20, 21). This may be because Hong Kong culture is strongly influenced by Chinese collectivism, where people are prone to considering young children with DD as bad seeds and a disgrace to their families (22, 23). Consequently, parents of young children with DD may experience stigma, which could lead them to withdraw themselves and their children from community participation (24). These findings highlight the need to support Hong Kong parents and their children and promote community participation.

In Hong Kong, early intervention services are available to young children with DD and are mainly offered through early education and training centers, special child care centers, integrated programs in kindergarten-cum-child care centers, or on-site pre-school rehabilitation services (25). Depending on individual needs, these services may provide children with direct interventions such as occupational therapy, physiotherapy, and/or speech therapy. Parents of young children with DD may also be provided parent training on child development, childcare, and parenting skills. While early intervention services were proven to improve children's functional skills (26, 27), emerging evidence has indicated that interventions targeting skills-based components do not confer participation gains as expected (28). A recent study identified a positive relationship between the intensity of early intervention services and children's participation in home-based activities (29). However, factors influencing participation in community activities appear to be more complicated than those in home activities (5, 6, 30), because community participation involves other resources and people in the community as well as environment in which the activities take place. In addition to existing early intervention services, other services are needed to promote the community participation of children with DD under the age of seven in Hong Kong.

In recent years, parent coaching has gained international interest as an individually tailored approach for increasing the participation of children with disabilities (31, 32). Occupational performance coaching (OPC) (33) is a seminal coaching model developed for children with DD. OPC directly targets children's participation in the living environment by working with parents as mediators of change for their child. It includes specific techniques to heighten parents' engagement in the action-reflection coaching process, thus optimizing the conditions for change. Through coaching, parents learn to develop their problem-solving ability by identifying novel, ambitious, but highly individualized, and directly applied strategies to improve their child's participation. As such, OPC takes an enablement-focused, family-centered, and ecologically oriented approach (33). OPC is then suitable for use with young children with DD to address their participation difficulties resulting from the multi-system complexity of their disability, including child, family, and environmental influences.

The theoretical foundation of OPC is grounded in self-determination theory, which posits that three basic psychological needs drive intrinsically motivated behavior: autonomy, relatedness, and competence (34). *Autonomy*, the need to feel that behavioral choices are aligned with personal values and goals, is supported in OPC by inviting parents to direct goal-setting conversations, initiate analysis of goal situations, and select actions intended to improve goal progress. *Competence*, the need to be and feel competent in actions, is met during OPC by guiding parents to select actions that they perceive as achievable and likely enhance children's participation. *Relatedness*, the need to feel connected and accepted by others, is explicitly cultivated during OPC to enhance the conditions for trust and disclosure. Collectively, the fulfillment of these psychological needs is predicted to elevate parents' intrinsic motivation to enact strategies and actions that contribute to their children's improved participation.

OPC has been shown to be potentially effective in increasing children's participation in various life situations (35–39). One of the first studies was an exploratory case study in Australia (36), in which three mothers of 5- to 9-year-old children experiencing difficulties in adaptive skills participated in 10 OPC sessions. After the intervention, the mothers reported change scores of 2–7 points for goal performance related to their child's participation in home and community activities, which exceeded the established minimal, clinically significant level of two points for the outcome measure used (40). Later, a pre-post study involved 29 Australian mothers of 5- to 12-year-old children with adaptive skills below the developmental age and identified a significant improvement in goal performance for their children's participation after receiving eight OPC sessions (35). This improvement had a large effect size and was particularly maintained at the 6-week follow-up. Recently, Kahjoogh et al. (39) conducted a randomized controlled trial (RCT) of OPC in 30 Iranian mothers of 5- to 11-year-old children with cerebral palsy. Children in the OPC intervention group exhibited significantly increased participation-related goal performance with a large effect size, compared to children in the control group. Several RCT studies are being conducted (41), which will provide cumulative evidence for the effect of OPC on children's participation in different populations or countries.

While the above-mentioned studies provided promising results regarding the effectiveness of OPC, most goals identified by the parents in those studies were related to self-care, and no separate analyses were conducted to evaluate its unique effect on community participation goals. Moreover, those studies did not employ specific participation measures, making it difficult to determine whether the improvement in individual goals after OPC could benefit children's overall community participation. Therefore, we conducted a case study of four parents of young children with DD aged 4–5.5 years in Hong Kong to evaluate the feasibility of OPC in Chinese culture and its effect using individualized and community participation measures (42). This study found a trend of improvement in community participation goal performance and child involvement in community activities. However, the findings of the case study provide only preliminary evidence of the effect of OPC on children's community participation.

To demonstrate the effect of OPC on children's community participation, a full-scale RCT that compares the OPC with a component-equivalent control intervention is needed. Pilot studies that evaluate the feasibility of key study components and obtain an initial estimate of the effect are the first step to increase the likelihood of success in running a fully powered and efficacious RCT of OPC (43). Therefore, it is necessary to know which intervention and assessment protocol (e.g., recruitment, retention, adherence, blinding success, and fidelity) is feasible for conducting the RCT of OPC with parents of young children with DD in Hong Kong. It is also valuable to obtain preliminary evidence for the efficacy of OPC, relative to certain control treatment, specifically in the areas of child community participation. Additionally, the experiences of parents and therapists in Hong Kong who deliver OPC during the coaching sessions can be studied to provide insight into the acceptability of OPC in local contexts. To advance this knowledge, a Phase 1 RCT is needed. In this paper, we propose a study protocol that will be used in the Phase 1 RCT which aims to evaluate the feasibility of conducting an RCT of OPC in Hong Kong, the acceptability of the coaching intervention, and the initial efficacy of OPC on promoting children's community participation.

## Materials and Methods

### Trial Design

**Figure 1:**
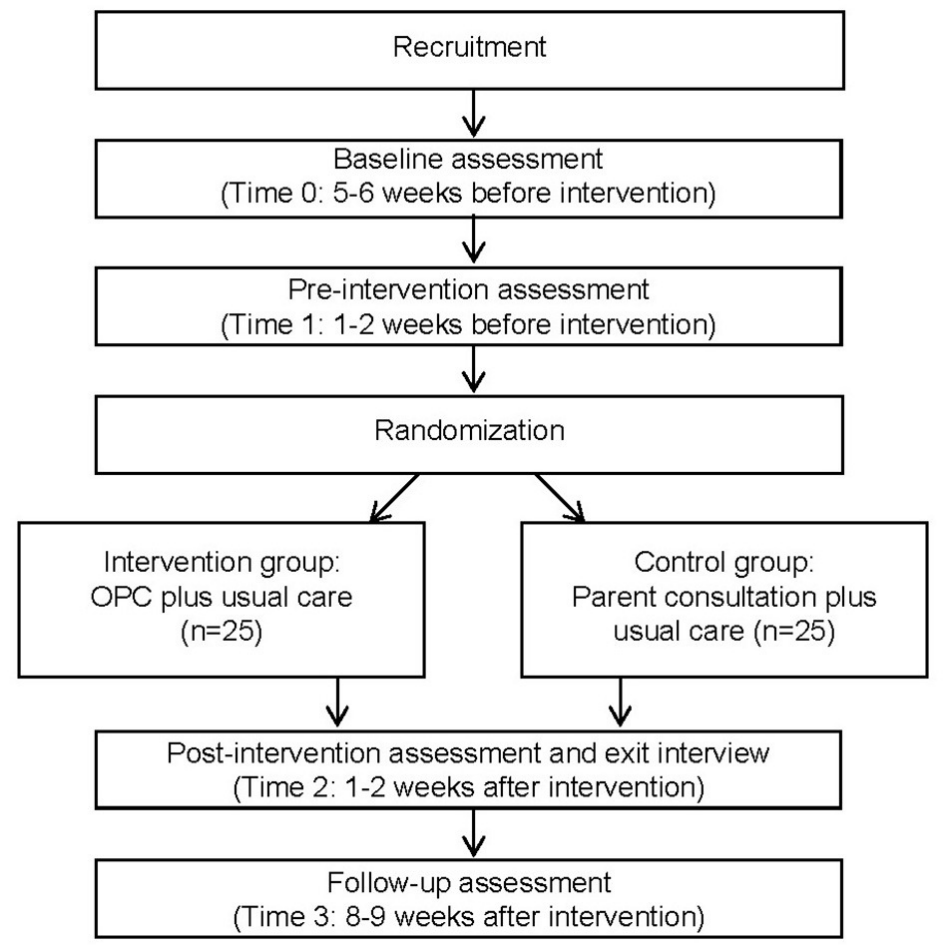
Flowchart of data collection. OPC, Occupational Performance Coaching.

We propose a two-arm parallel, double-blind design for this Phase 1 RCT of OPC. Parents of young children with DD will be randomly assigned to the intervention group (receiving OPC), and the control group (receiving parent consultation) and will be blinded to the group type that they are assigned to. Parent consultation is chosen as the component-equivalent control treatment, because it is a common approach used by rehabilitation therapists to improve children's adaptive behavior and parenting skills (44). Meanwhile, both groups will continue to receive usual care during the study period. The trial design is illustrated in Figure 1. The present protocol was prepared according to the recommendation for good practice in RCT feasibility and pilot design (43, 45).

### Study Setting and Participants

The RCT will be conducted in Hong Kong across three major geographical regions (Hong Kong Island, Kowloon, and New Territories). The target population will comprise families with young children awaiting or receiving early intervention services. To be eligible for the study, participants will have to (a) be one/both parent(s) of a child aged 2–6 years old who have been clinically diagnosed with a DD (including but not limited to intellectual disability, developmental delay, or autism spectrum disorder) given by pediatricians/psychiatrists; (b) be the child's main caregiver who has a long-term parenting role with at least 50% of caregiving responsibilities; (c) be able to converse in Chinese; and (d) have the desire to improve their child's participation in four community activities that are selected from the Young Children's Participation and Environment Measure (YC-PEM, detailed later).

Participants will be excluded if their child has DD combined with physical impairment (e.g., amputation, cerebral palsy, spina bifida) or sensory impairment (e.g., blindness, deafness). This is because the support and resources needed to improve community participation for these children may differ from those for children with DD without physical/sensory constraints.

### Sample Size

The sample size was calculated using Morgan and Case's formula (46) with the following set-up: type I error of 0.05, power of 0.90, 1-to-1 random allocation, and variance ratio of 0.44, using a conservative assumption for the compound symmetry correlation structure. A large effect size (Cohen's *d*) of 0.80 was determined based on the pooled effect sizes derived from our previous study (42) and a recent RCT (39). Therefore, a minimum sample size of 30 participants will be required in this pilot RCT to test for 2 × 3 mixed design analysis of covariance (i.e., the number of two groups with three repeated measurements, adjusted for individual differences in baseline assessments). Allowing for an attrition rate of 40% that was observed in the previous study (42), a total of 50 parent-child dyads (25 in each group) will be recruited.

### Recruitment Method

Participants will be recruited from early intervention services within three non-governmental organizations in Hong Kong. Occupational therapists who work in each service will assist in the initial screening of potentially eligible families of children receiving services. They will then provide the parents of interest with the study information sheet and consent form. Once the signed consent forms are returned, a research assistant who oversees the trial will contact parents by phone to further screen for study participation eligibility. Posters and social media will be used to recruit families of children who are awaiting early intervention services. Parents of interest will be asked to contact the same research assistant for screening and, if eligible, to complete the consent forms.

### Randomization and Blinding

Block randomization stratified by engagement in early intervention services (awaiting vs. receiving) based on the 1-to-1 allocation ratio will be used to assign participants to the intervention or control group. The randomization sequence will be computer-generated, and allocation will be completed by another research assistant not associated with the study. Participants and independent outcome assessors will be blinded.

### Intervention and Control Treatment

#### Intervention Treatment: Parent Coaching

The OPC intervention comprises three components defined as the enabling domains: (1) connect–building parents' trust in the coach by using verbal and nonverbal strategies such as listening, empathizing, and partnering; (2) structure–building parents' competence by adopting a problem-solving framework of setting goals, exploring options, planning action, carrying out plans, checking performance, and generalizing; and (3) share–building parents' autonomy by reciprocally exchanging information between the coach and parents with an emphasis on eliciting parents existing knowledge. In particular, collaborative performance analysis is used to explore the options for a particular goal. In this collaborative performance analysis, the coach follows four steps: (a) identify parents' perception of what currently happens, (b) identify what they would like to happen, (c) explore barriers and bridges to the desired performance, and (d) identify their needs to take actions to achieve goals. Throughout these steps, parents are guided to find strategies to facilitate their children's performance in order to support goal achievement.

In this pilot RCT, we propose that the OPC intervention will consist of four to eight weekly (or fortnightly) sessions in correspondence with the number of goals identified by parents and the progress of the goal achievement. Each session will last 30 min to 1 h. Depending on parents' needs, coaching sessions will be delivered in person with one or both parent(s) in therapeutic/office rooms located at participating early education and training centers, special child care centers, university campuses, or *via* telephone or other communication applications (e.g., Zoom or WhatsApp). Parents will be allocated to the same coach throughout the intervention period, and the coach will not be the treating therapist of their child. Because OPC focuses on coaching parents, children's attendance at the coaching sessions will be at the parents' discretion.

Coaches who deliver OPC will be occupational therapists working in participating non-governmental organizations who have at least 2 years of experience working with children/parents. A total of 29 therapists attended a 16-h online training workshop delivered by the OPC developer (i.e., the last author) in March 2020. The workshop involved the translation of coaching techniques to participants using case examples, video, live demonstrations, role play, discussion, and active planning for implementation in specific practice settings. Further, 14 of the therapists attended a 4-h follow-up training by the OPC developer in May 2020, and eight of them were mentored for various hours relating to intervention fidelity by the first author, who is a qualified OPC trainer. In total, the training for each coach was at least 24 h cumulatively, and they will be dropped if they do not demonstrate ≥80% fidelity in the practice of one real case prior to study commencement. This is the minimum requirement recommended in the OPC manual (47) for conducting related research projects. Once the intervention begins, the first author will provide the coaches with continuous supervision and mentoring through individual meetings and/or Google forums when their self-rated fidelity of OPC in any sessions does not achieve 80%. All coaching sessions will be audio-recorded to monitor intervention fidelity.

#### Control Treatment: Parent Consultation

Parents who are randomized to the control treatment will receive consultation regarding community resources from occupational therapists or occupational therapy students who are not involved in OPC training or meetings in the study. A toolbox of community resources has been developed by the research team by identifying public playgrounds, play groups, and sports programs sponsored by non-governmental organizations or government from the website of the Leisure and Cultural Services Department (www.lcsd.gov.hk/en/). It also included generic supportive strategies for parents of children with disabilities, as drawn from the existing literature (30, 48, 49). Occupational therapists or master of occupational therapy students who have studied rehabilitation psychology and fundamental occupational therapy subjects will use the toolbox to provide parents with available environmental resources and strategies to enhance the community participation of their child with DD, followed by an understanding of the current situation and the identification of problems encountered by parents. The direct informing approach (50) will be used to instruct parents about the availability of environmental resources close to their living areas and what they can plan to do by using possible supportive strategies. In addition, information about child disability and/or developmental milestones may be provided if needed. However, the OPC key elements, such as parents' involvement in the action-reflection process and collaborative performance analysis will be avoided in the consultation.

The consultations will be conducted for four to eight weekly/fortnightly sessions depending on the parents' needs, and each session may last 30 min to 1 h. The consultations will be delivered in person or in tele-format at the parents' discretion. Prior to the study, occupational therapists and occupational therapy students will be trained by the first author in the use of the toolbox. A 2-h training session will be held including the introduction of strategies and resources included in the toolbox and the procedure to provide consultation, followed by role-play practice. They will be supervised regularly in monthly meetings throughout the study period. Parents will be allocated to the same trained therapists or students for consultation during the study period.

#### Usual Care

Children who are randomized to either the intervention or control group will continue to receive usual care. Depending on individual needs or status, usual care may include (a) waiting to access early intervention services; (b) services provided by the training/care centers, such as occupational therapy, physiotherapy, and speech therapy on a weekly/monthly basis; and (c) private therapy. Of the early intervention services, occupational therapy in Hong Kong focuses on improving children's fundamental skills (e.g., fine motor, sensory integration, visual perception, and pre-writing) and self-care abilities—mostly through direct training on children. Thus, it will have a minimal effect on children's community participation. To understand the variability in usual care received by children between the intervention and control groups, parents will be asked to complete a therapy-activity log during the study period, which will record the type(s) and duration of service(s) children receive on a weekly basis.

### Outcome Measures

#### Assessment Timing

This pilot RCT will use four assessment points similar to the design of Graham et al.'s study (35). The four time points are: 5–6 weeks before intervention (time 0 for baseline assessment), 1–2 weeks before intervention (time 1 for pre-intervention assessment), 1–2 weeks after intervention (time 2 for post-intervention assessment), and 8–9 weeks after the intervention (time 3 for follow-up assessment). Table 1 outlines outcome measures that will be administered at each assessment point.

**Table 1:** Outcome measures used and timing in the study.

**Outcome measure**	**Method**	**Variable(s)**	**Time**	**Assessment timing**				
				**Baseline** **(Time 0)**	**Pre-OPC** **(Time 1)**	**OPC**	**Post-OPC** **(Time 2)**	**Follow-up (Time 3)**
Demographics	Parent-report questions	Gender, age, diagnosis, family income and structure, having a domestic helper, parents' information (age, education), number of services used	5 min	√				
PEDI-CAT (64)	Parent interview	Scaled scores for each of two domains (daily activity and social/cognitive function)	10–15 min	√	√		√	√
COPM (40)	Parent interview	Performance and satisfaction scores for each community-related participation goal(s)	20 min	√	√		√	√
YC-PEM (53)	Parent-report questions	Average scores for each of three dimensions (participation frequency, participation involvement, and environmental support) in the community setting	15 min	√	√		√	√
DASS-21 (60)	Parent-report questions	Total scores for each of three subscales (anxiety, depression, and stress)	10 min	√	√		√	√
Kiddy-KINDL (61)	Parent-report questions	A grand total score of four domains (emotional well-being, self-esteem, family, and social contact)	10 min	√	√		√	√
PSOC (59)	Parent-report questions	Total scores for each of two dimensions (satisfaction and efficacy)	10 min	√	√		√	√
HCCQ (69)	Parent-report questions	An average score for perceived autonomous supportiveness from coaches	5 min				√	
PGIC (72)	Parent-report question	A 7-point Likert score of overall improvement in child community participation	<2 min				√	√
*Post-hoc* guess for treatment assignment	Parent-report question	A binary choice of parent coaching or parent consultation that parents receive	<2 min				√	
SRS (68)	Parent-report questions	A total score for working alliance during each coaching session	5 min			√		
OPC FM Version 3 (47)	Coach-report questions	A percentage score for coaching fidelity during each session	10 min			√		

*OPC, Occupational Performance Coaching; PEDI-CAT, Pediatric Evaluation of Disability Inventory-Computer Adaptive Test; COPM, Canadian Occupational Performance Measure; YC-PEM, Young Children's Participation and Environment Measure; DASS-21, Depression Anxiety Stress Scales-21 items; PSOC, Parenting Sense of Competence Scale; HCCQ, Health Care Climate Questionnaire; PGIC, Patient Global Impression of Change; SRS, Session Rating Scale; OPC FM, Occupational Performance Coaching Fidelity Measure*.

#### Study Assessments

##### Objective 1: Feasibility of the Trial

The feasibility of the trial will be evaluated using five indicators (recruitment, retention, adherence, blinding success, and fidelity) with predetermined criteria, as shown in Table 2. In particular, the OPC Fidelity Measure Version 3.0 (47) will be used by the first author to rate the audio recordings of the eight selected coaching sessions to verify the intervention fidelity of each coach. The eight sessions that will be selected will include the first two participants' first two sessions and then four randomly selected sessions from the remaining sessions of the first two and other participants who are coached by the coach.

**Table 2:** A priori success criteria to assess the feasibility of the trial.

	**Definition**	**Success criteria**
Recruitment	Percentage of eligible families agreeing to participate in the study	≥20% recruitment response rate achieved
Retention	Percentage of participants who complete the trial	≥60% retention rate achieved (i.e., completion of all assessments)
Adherence	Percentage of coaching sessions attended by parents in the intervention group	≥75% adherence rate achieved, based on our previous research of similar duration (42)
Blinding success	Percentage of parents who guess treatment allocation correctly after the study	50% based on the guess of treatment by chance (50/50)
Fidelity	Degree to which the OPC is implemented by coaches as intended	≥80% fidelity on the OPC Fidelity Measure Version 3.0 in eight selected session per coach

*The OPC Fidelity Measure Version 3.0 consists of 18 items across five domains: relationship, goal, reflection, analysis and action, client response, and distinguishing. Each item is rated on a three-point Likert scale (1 = low and 3 = high). The percentage score will be calculated by dividing the total score by the possible maximum score. Higher percentage scores indicate higher fidelity, and the cut-off of sufficient fidelity of OPC per session is set at 80%*.

##### Objective 2: Acceptability of OPC

The acceptability of the OPC intervention will be assessed through semi-structured interviews with parents at time 2 (i.e., 1–2 weeks after the intervention) and with coaches at the end of the study. Parents will be asked about their satisfaction with the coaching sessions (e.g., relationship with the coach, schedule, and duration), experience in being coached (e.g., what they have learned, what they like most/least, and the challenges experienced), and the perceived impact of OPC on children's participation in community activities. Coaches will be interviewed to evaluate their experience of delivering OPC intervention (e.g., perceived effectiveness, challenges, optimal coaching schedule/duration, and opinions on cultural suitability).

##### Objective 3: Initial Efficacy of OPC

###### Primary Outcome Measures.

***Canadian Occupational Performance Measure***. The COPM (40) will be used to measure parents' perceptions of children's participation in specific community activities. This measure is selected for use because it can identify individualized problems in participation in occupations and then help to formulate goals related to child participation through semi-structured interviews. In the interview, parents are further prompted to rate their child's performance and their satisfaction with the current status on a 10-point Likert scale (1 = *not good/satisfied at all* and 10 = *optimal performance/satisfaction*). High scores indicate greater children's participation performance and parents' satisfaction. In this pilot RCT, we propose that parents' identified goals will not be limited to the community participation but will also be extended to other life areas. An adequate internal consistency (Cronbach's α = 0.73–0.88) of the COPM has been reported (51). The prioritized problems using the COPM in parents of children with disabilities were also found to be corresponding with specific items in the Pediatric Evaluation of Disability Inventory, demonstrating construct validity of the COPM (52).

***Young Children's Participation and Environment Measure***. The YC-PEM (53) will be used to capture children's overall community participation patterns. This measure is selected because it is a parent report questionnaire that can be used for young children with various disabilities. The YC-PEM also has a community section that includes 11 participation items across four broad categories of neighborhood and community outings, classes and groups, community-sponsored activities, and recreational activities and trips. In each item, parents are asked to rate: (a) how often their child has participated in the past 4 months using an 8-point Likert scale (0 = *never* and 7 = *once or more each day*); (b) how involved the child is during participation using a 5-point Likert scale (1 = *not very involved* and 5 = *very involved*); and (c) parental desire for change in the child's participation (yes/no and, if yes, six nominal options for the type of desired change can be selected). Total scores are generated by averaging all items in the participation frequency and involvement dimensions. High scores indicate greater children's participation frequency and involvement. The YC-PEM participation scale has acceptable internal consistency (α = 0.64–0.78) and test-retest reliability (intraclass correlation coefficients [ICC] = 0.82–0.89) (53–56). Moreover, it demonstrates known-group validity between children with and without disabilities (21, 53, 57, 58) and convergent validity by correlating with functional performance of children with disabilities (21, 53).

###### Secondary Outcome Measures.

***Parenting Sense of Competence Scale***. The PSOC (59) is a parent report questionnaire to obtain parents' perceptions of their parenting role, and this scale is selected because it will help to examine whether parents who receive OPC will have improved parenting competence. The PSOC has two dimensions: efficacy (eight items) and satisfaction (nine items). Parents are asked to rate each item on a 6-point Likert scale (6 = *strongly disagree* and 1 = *strongly agree*). Total scores are generated by summing all items in each dimension (after reversing the scores of some items). High scores indicate greater competence and satisfaction with parenting, respectively. The PSOC has demonstrated good internal consistency (α = 0.77–0.80) and test-retest reliability (ICC = 0.82–85) (59).

***Depression, Anxiety, and Stress Scale-21***. The DASS-21 (60) is a self-report questionnaire that includes 21 items assessing people's negative emotional states of depression, anxiety, and stress (seven items in each subscale). In the proposed RCT, the use of the DASS-21 is determined because it can be completed by parents to provide insight into the beneficial impact of OPC on promoting parents' emotional states. In the DASS-21, each item is rated on a 4-point Likert scale (0 = *did not apply to me at all* and 3 = *applied to me very much or most of the time*). Total scores are generated by summing all items in each subscale, with high scores indicating greater emotional problems. Good internal consistency (α = 0.77–0.87) of the DASS-21 has been reported (60).

***KINDL Questionnaire***. This questionnaire measures health-related quality of life in children and has three age versions with both child and parent reports, including Kiddy-KINDL for parents of children aged 3–6 years (61). Because self-report is difficult for young children with DD, the parent-report version of Kiddy-KINDL is determined for use in the proposed RCT to explore whether children have improved psychosocial health after the OPC intervention. The Kiddy-KINDL comprises 24 items that assess parents' perceptions of their child's health-related quality of life across physical well-being (four items), emotional well-being (four items), self-esteem (four items), family (four items), social contacts (four items), and school functioning (four items). The recall period covers the last month in this study, and each item is rated using a 5-point Likert scale (0 = *never* and 4 = *all the time*). A psychosocial health score is generated by summing item scores from the emotional, self-esteem, family, and social contacts domains (62). High scores indicate greater psychosocial health. The Kiddy-KINDL has demonstrated acceptable internal consistency (α = 0.70–0.89) (61, 63).

###### Other Exploratory Measures

***Demographic questionnaire***. In the proposed RCT, we will design a parent-report questionnaire to collect demographic information such as child age and gender, family structure, family income, employment of a domestic helper, as well as parents' age, and education. Parents will also be asked to report the type(s) of clinical diagnosis or disability which their child has and rate the severity of their child's DD as a whole using a 4-point Likert scale (1 = *very mild* and 4 = *severe*). The demographic and clinical information will be used to characterize children and their parents in the intervention and control groups for comparison.

Additionally, we consider that the availability of early intervention services may have an effect on children's functional performance and participation based on literature (27, 29). Therefore, this questionnaire will also ask parents to tick the type(s) of early intervention service(s) their child has received in the past month, including occupational therapy, speech therapy, physical therapy, and center-based training which are common early intervention services in Hong Kong. The number of the service use will be categorized and used as a control variable adjusted for baseline differences in the analysis of the efficacy of OPC.

***Pediatric Evaluation of Disability Inventory Computer-Adaptive Tests***. The PEDI-CAT (64) is a parent-report, computer-based assessment of children's functional performance across four domains: daily activity, social/cognitive function, mobility, and responsibility. In the proposed RCT, we will select the use of the first two domains, operated by the speedy feature, for the purpose of exploring the improvement in children's daily activities and social/cognitive function. The speedy feature allows reducing administration time by selecting suitable 10–15 items to assess based on the relative difficulty of preceding items and parents' responses to those items (instead of completing a full set of items). In each PEDI-CAT item, parents rate their child's typical performance using a 4-point Likert scale (1 = *unable* and 4 = *easy*). Scaled scores of each domain are derived based on the estimates of the placement of individual children along the hierarchical scales that have been calibrated using item-response theory in the standardization samples (64). The PEDI-CAT scaled scores are on a 20–80 metric and have been recommended for use to evaluate changes over time (64). The PEDI-CAT has demonstrated excellent agreement with the full-length version (Pearson's *r* = 0.94–0.99) (64, 65) and satisfactory test-retest reliability (ICC = 0.86–0.92) (64, 66).

***Environmental Support Scale of the YC-PEM***. In the community section of the YC-PEM mentioned earlier, parents will also be asked to evaluate the impact of the types of environmental features (10 items) and resources (seven items) regarding their child's participation in community settings. This scale is selected for use because we would like to explore whether parents who receive OPC have higher perceived environmental support for their children's community participation. In the environmental support scale, a 3-point Likert scale is used to assess the level of parents' perceived impact of environmental features (1 = *usually makes it harder* and 3 = *no impact/usually helps*) and resources (1 = *usually no* and 3 = *not needed/usually yes*) on participation, respectively. Total scores are generated by averaging all items on this scale, with high scores indicating greater environmental support. This environmental support scale has acceptable internal consistency (α = 0.83) and test-retest reliability (ICC = 0.78) (21, 53).

***Session Rating Scale***. The SRS is a four-item visual analog scale that assesses therapeutic alliance at the end of each session, and this scale is used because it provides insight into the potential mechanism of parents' intrinsic motivation to enact actions during OPC. Each of the four items captures a key dimension of effective therapeutic relationships, including respect and understanding, relevance of the goals and topics, approach used in therapy, and overall alliance. Parents are asked to place a mark on a 10-cm line nearest the pole that best describes their experience with their OPC coach. Total scores are generated by summing up the marks made by parents measured to the nearest centimeter on each of the four lines. Higher scores indicate greater therapeutic alliance. The SRS has been reported to be internally consistent (α = 0.88–0.96) and reliable over time (*r* = 0.63) (67, 68).

***Health Care Climate Questionnaire***. The HCCQ assesses people's perceptions of health care practitioners' autonomy support in a given program grounded by self-determination theory (69). It consists of 15 items rated on a 7-point Likert scale (1 = *strongly disagree* and 7 = *strongly agree*). One example item is “I feel that my health-care practitioner has provided me with choices and options about regular exercise.” Total scores are calculated by averaging all item scores, with higher scores indicating greater autonomy support. The HCCQ has been adapted by Chan et al. (70) for use in the physiotherapy context by replacing “health-care practitioner” with “physiotherapist” and eliminating the statement of the specific program (e.g., regular exercise). In the proposed RCT, we will adopt Chan et al.'s version with a slight amendment of the wording to “my coach.” The modified version of the HCCQ will allow us to understand parents' perceptions of the degree to which their coach is autonomy supportive (vs. controlling) in coaching them regarding their child's participation. The HCCQ has demonstrated acceptable internal consistency (α = 0.94–0.95) in various studies (69–71).

***Patient Global Impression of Change***. This measure is proposed for use, because it can provide an indicator of parents' global impression of whether their child's participation in community activities has been better, about the same, or worse since the start of the given intervention. The PGIC includes only one item that is scored on a 7-point Likert scale (1 = *very much improved* and 7 = *very much worse*). This measure has been reported to demonstrate good clinimetric properties (72).

### Data Analysis

Descriptive statistics will be used to characterize the participants and evaluate the trial feasibility according to our a priori success criteria. The *t*-test (or Mann-Whitney U test) and chi-square statistics will be used to test for between-group baseline differences. Prior to the efficacy analyses of OPC, the normality of the data for the studied variables will be examined and, if the data are not normally distributed, transformation methods will be applied.

To evaluate the acceptability of OPC intervention, interviews with parents and coaches will be transcribed and then analyzed separately using qualitative methods. Specifically, thematic analysis using a data-driven inductive approach (73) will be used to scrutinize the parents' interview transcripts and interpret their coaching experience as well as perceived impact. Thematic analysis is chosen, because it provides a flexible method for identifying, analyzing, and reporting patterns (themes) within data without a prior coding scheme (74). Alternatively, coaches' interview transcripts will undergo conventional content analysis (75) to describe their experience in delivering OPC intervention. We choose content analysis because only eight coaches will be involved in the proposed RCT and data saturation may not be achieved if thematic analysis is used. Two research team members will be involved in the thematic and content analyses by following the recommended procedure (76). To establish the trustworthiness of the thematic and content analyses, code-recode, peer checking, and team discussions will be used (77).

To evaluate the initial efficacy of OPC on the primary and secondary outcomes, we will use the repeated-measures analysis of covariance (ANCOVA) by controlling for baseline variability at time 0 as well as the variations in treatment dosage and delivery format (e.g., in person or tele-format). That is, the repeated-measures ANCOVAs will be used to compare the change in the scores of outcome measures across the three time points (at times 1, 2, and 3) by controlling for baseline differences and treatment variations. For participation goals identified by parents in the COPM, only community-related participation goals will be targeted for analysis. Principles of intent-to-treat analysis will be applied and, if participants withdraw after the coaching/consultation sessions, their data for subsequent time points will be imputed by carrying the last assessment forward. *Post-hoc* analyses using the Schffé method will be performed when the main comparison results are significant. Statistical significance will be set at *p* < 0.05. Estimates of effect sizes with 95% confidence intervals will be calculated for each outcome measure.

## Discussion

In this paper, we have presented the study protocol for a pilot RCT that will evaluate the feasibility and acceptability of delivering a parent-coaching intervention (i.e., OPC) in Hong Kong. The pilot RCT will also test the efficacy of OPC, relative to parent consultation, in promoting community participation among young children with DD. The OPC, grounded in self-determination theory, aims to work with parents to recognize and implement changes in the living environment to support participation performance for themselves and their children (33). The OPC is distinct from parent consultation in which professionals act as experts to instruct parents on how to apply strategies and/or obtain resources (32). The OPC is a family-centered, goal-directed, and ecologically oriented intervention through coaching parents (33) and, therefore, it is possible to address children's participation difficulties through OPC. Despite an increasing number of studies of OPC (35–39, 42), there is a lack of high quality evidence on its effect specific to community participation. Community participation is not the primary focus of current early intervention services in Hong Kong. We believe that this is the right time to conduct a pilot RCT of OPC. This pilot study will be the first step in determining whether the OPC intervention and our proposed study protocol are feasible and acceptable for conducting a full-scale RCT. The results of the pilot RCT will also inform future preparation for conducting a definitive efficacy trial of OPC, with the aim to increase the likelihood of success and confidence in the findings.

To our knowledge, this is the first study describing a RCT protocol of OPC with specific focus on primary outcome related to community participation. In past RCTs of OPC (39, 41), the COPM has been commonly used to measure child performance and parents' satisfaction with individualized goals in the areas of self-care, productivity, and leisure. As goals could be varied across each family, we plan to prompt parents to set as many goals related to their child's community participation as possible, and singe out those goals for analysis in our pilot RCT. Furthermore, we propose using the community section of the YC-PEM. The YC-PEM is a contemporary measure that can evaluate participation patterns (frequency and involvement) of 11 typical community life situations, together with parent-perceived environmental support, in children aged younger than 6 years (53). It is anticipated that, by using both the COPM and YC-PEM, our pilot RCT can provide preliminary evidence on whether OPC leads to beneficial effects on children's community participation at both individual goal level and overall participation level. These results will help estimate effect size that can be used in sample size calculation required to power subsequent RCTs.

Secondary outcome measures such as the DASS-21 and Kiddy-KINDL will be included in the proposed pilot RCT. This will help determine whether the effect of OPC on goal achievement could translate into improvement in parents' emotional states and their child's health-related quality of life. The improvement in these areas is crucial given that disability could impede children's participation, which in turn affect quality of life and parents' emotional status (8, 10, 11). Previous case studies found that, after OPC, parents developed insights about their children's difficulties and learnt strategies to improve the children's physical wellbeing (42). The parents also demonstrated less stress, anxiety, and depression as they felt understood and supported by coaches (36, 42). While the extended effect of OPC on health-related outcome is promising, it is necessary to be supported by high quality evidence (e.g., the currently proposed pilot RCT).

The results of the proposed pilot RCT will shed light on the potential mechanism of action; that is, parents' self-determination derived from satisfaction of three psychological needs of autonomy, competence, and relatedness. Among the three needs, parents' increased competence has been identified in most studies examining the effect of OPC (35, 37, 39, 42). By contrast, the needs for autonomy and relatedness were seldom studied. Hence, we incorporate the HCCQ and SRS as exploratory measures to assess autonomy supportiveness and working alliance, respectively. Together with parents' self-competence that will be captured by the PSOC as secondary outcome measure in the pilot RCT, this study will help us to explore whether the fulfillment of the three psychological needs could work as the mechanism of change in OPC.

While the study protocol is planned carefully, there are several potential pitfalls. First, parents will set goals prior to the intervention. As the goal-setting process may have beneficial effects, both groups of children may show improvement in community participation afterward. Therefore, we will examine whether there is an improvement between baseline and pre-intervention and, if there is, the baseline difference (or the change) will be accounted for in subsequent efficacy analysis. Second, parents who expect expert consultation may be prone to declining the OPC intervention after the first few sessions. This situation is unavoidable as it has happened in our pilot case study (42). The withdrawal will result in an unbalanced number of subjects in the intervention and control groups and further affect the analysis. To mitigate the uneven withdrawal effect, two-level withdrawal will be structured and sought for parents' consent. These are, withdrawal from the OPC intervention only but continuing in the trial for follow-up, or complete withdrawal without further contact. Lastly, the coronavirus disease 2019 pandemic has led to repeated implementation of disease containment measures, which may limit children's participation in community activities. To avoid contaminating the trial, we will closely monitor the announcement of these measures and take necessary actions such as suspension of recruitment or early termination followed by immediate post-intervention evaluation.

## Ethics Statement

The feasibility and pilot RCT of OPC has been approved by the Human Subjects Ethics Subcommittee at the Hong Kong Polytechnic University (reference number: HSEARS 20190114005-1). Written and informed consent will be obtained from parents of young children with DD prior to participation in the trial.

## Author Contributions

C-WC, YL, C-YL, and FG contributed to the conceptualization and methodology design. C-WC initially drafted the manuscript. YL, C-YL, and FG critically revised the manuscript. All authors have read and agreed to the published version of the manuscript.

## Funding

This study was funded by the Health and Medical Research Fund, the Food and Health Bureau, the Government of the Hong Kong Special Administrative Region (Grant Reference Number: 02180358).

## Conflict of Interest

The authors declare that the research was conducted in the absence of any commercial or financial relationships that could be construed as a potential conflict of interest.

## Publisher's Note

All claims expressed in this article are solely those of the authors and do not necessarily represent those of their affiliated organizations, or those of the publisher, the editors and the reviewers. Any product that may be evaluated in this article, or claim that may be made by its manufacturer, is not guaranteed or endorsed by the publisher.
